# A pictorial review of the pathophysiology and classification of the magnetic resonance imaging patterns of perinatal term hypoxic ischemic brain injury – What the radiologist needs to know…

**DOI:** 10.4102/sajr.v24i1.1915

**Published:** 2020-10-30

**Authors:** Shalendra K. Misser, Anthony J. Barkovich, Jan W. Lotz, Moherndran Archary

**Affiliations:** 1 Department of Radiology, Faculty of Health Sciences Medicine, College of Health Sciences, Nelson R. Mandela School of Medicine, University of KwaZulu-Natal, Durban, South Africa; 2 Department of Radiology, Faculty of Radiology, Lake, Smit and Partners Inc, Durban, South Africa; 3 Department of Radiology, Faculty of Medicine, Neurology and Neurosurgery, Division of Neuroradiology, University of California, San Francisco, United States of America; 4 Department of Radiology, Faculty of Medicine, University of Stellenbosch, Stellenbosch, South Africa; 5 Department of Paediatrics, Faculty of Health Sciences Medicine, College of Health Sciences, Nelson R. Mandela School of Medicine, University of KwaZulu-Natal, Durban, South Africa

## Abstract

This article provides a correlation of the pathophysiology and magnetic resonance imaging (MRI) patterns identified on imaging of children with hypoxic ischemic brain injury (HIBI). The purpose of this pictorial review is to empower the reading radiologist with a simplified classification of the patterns of cerebral injury matched to images of patients demonstrating each subtype. A background narrative literature review was undertaken of the regional, continental and international databases looking at specific patterns of cerebral injury related to perinatal HIBI. In addition, a database of MRI studies accumulated over a decade (including a total of 314 studies) was analysed and subclassified into the various patterns of cerebral injury. Selected cases were annotated to highlight the areas involved and for ease of identification of the affected substrate in daily practice.

**Keywords:**

Hypoxic ischemic encephalopathy; Magnetic resonance imaging; Acute profound; Partial prolonged; Hypoxic ischemic brain injury; Ulegyria; Multicystic; Encephalopathy.

## Introduction and background

Perinatal hypoxic ischemic brain injury (HIBI) is the leading cause of neonatal encephalopathy^1^ and accounts for between 6% and 8% of cerebral palsy worldwide.^2^ In developed countries, the prevalence is estimated at 1.5–4 per 1000 live births,^3^ whereas in developing countries, including South Africa, it is estimated to be much higher, up to 15.2 per 1000 births.^4,5^ Whilst therapeutic hypothermia has been shown to be beneficial, it is still not readily available in resource-limited settings; despite its use, the mortality and morbidity associated with HIBI remains high globally. By the age of 2 years, more than half of HIBI-affected children would have either not survived or suffered severe disabilities.^6^

In addition to the poor outcome and social impact of HIBI, there is grave concern internationally regarding the increase in medicolegal litigation in this aspect of medicine. The last decade has brought an explosion of cases against medical practitioners and institutions, particularly in children with cerebral palsy. In these cases, medical negligence is alleged and often the litigation is instituted a few, sometimes several, years after the delivery. Neuroimaging has evolved significantly in the last 30 years and plays an important role in the evaluation of children with suspected perinatal HIBI. Magnetic resonance imaging is the key imaging modality in the evaluation of suspected HIBI. As such, radiologists are increasingly being requested to lead pivotal evidence that may confirm a pattern of injury compatible with HIBI, and MRI has also been used in devising scoring systems for the evaluation of HIBI.^7,8^ The neuroimaging patterns of cerebral hypoxic ischemic injury have also been shown to be quite different in term neonates and preterm/premature neonates.^9^ This review pertains to the imaging of term neonatal hypoxic brain injury.

## Pathophysiology

**FIGURE 1:**
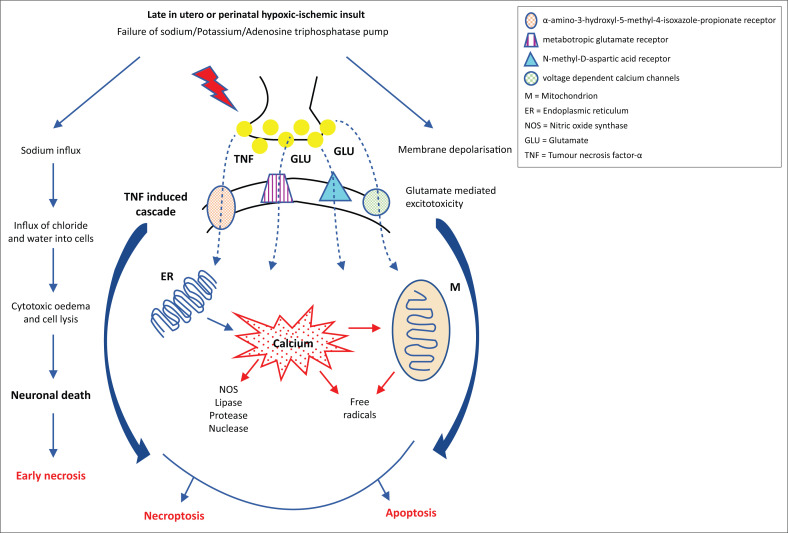
Pathogenesis of hypoxic ischemic encephalopathy^12^ mediated by sodium potassium adenosine triphosphatase pump failure leading to cytotoxic oedema and early cell necrosis, apoptosis via glutamate-induced excitotoxicity and necroptosis by tumour necrosis factor-α-induced cascade.

The basis of the injury demonstrated at MRI follows from the duration of the event preceding the hypoxic outcome, the severity of the hypoxia/hypoperfusion and the length of time for each of the subsequent phases of injury. There are three stages of injury in perinatal asphyxia.^10^ The first stage is the primary neuronal injury occurring immediately following reduction or absence of oxygen and/or glucose to the foetus. The primary cerebral energy failure causes adenosine triphosphate–dependent (ATP–dependent) sodium–potassium pump failure. This in turn results in cell swelling due to increased intracellular sodium and water. Through a cascade of secondary intracellular micro-events mediated by glutamate and tumour necrosis factor–alpha (TNF-∞), further cell death is the eventuality by apoptosis and necroptosis pathways, respectively (see Figure 1). In the second phase, there is a latent period which is variable, during which reperfusion occurs and some neurons can recover. In the subsequent 24 to 48 h, independent of the perinatal acidosis, secondary neuronal injury occurs due to dissemination of toxic neurotransmitters, largely because of reperfusion of affected brain regions.^11^ Tertiary injury evolves over the subsequent weeks to months with delayed neuronal necrosis as the injured brain undergoes remodelling and repair through astrogliosis.^12^

Critical to the evaluation of the MRI studies in a child with cerebral palsy is the ability to identify changes that would be attributable to a specific pattern of cerebral HIBI. It is possible to categorise the brain injury pattern based on the observed phenotype identified on neuroimaging. There have been several classifications noted in the literature for differentiating HIBI neuropathological imaging patterns. These, although named differently in multiple research studies, convey the same message in terms of the principal pathophysiology of injury demonstrable on MRI. For simplification, we propose classification into acute profound ischemia (API), partial prolonged ischemia (PPI), mixed patterns of ischemia and multicystic encephalomalacia (see Table 1).^13^ A similar classification of HIBI into diffuse, cortical/subcortical (cerebral cortex and deep nuclear pattern) and deep nuclear/brainstem MRI patterns correlates clinically with very severe/very prolonged, moderate to severe, prolonged/severe and abrupt insults, respectively.^14^ Consequent to the initial cause of injury, the resultant pattern of injury would depend on the ability of the foetal cerebral autoregulation mechanisms supporting brain perfusion. With regard to the multicystic encephalopathy or cystic encephalomalacia group, noting that there are several causes of this pattern of injury, we propose two subtypes that could result from hypoxic ischemic injury depending on the timing and severity of the insult. In most such cases, the entire cerebrum is affected, only sparing some portions of the temporal lobes. The key distinguishing feature is the involvement of the basal ganglia, which indicates probable primary (or superadded) acute profound ischemic injury.

**TABLE 1:** The basic patterns of magnetic resonance imaging abnormalities in hypoxic ischemic encephalopathy.

Subtype of HIBI	Anatomical structure involved	Timing and severity of insult
Acute profound ischemia	Deep nuclei/perirolandic/hippocampus	Sudden/profound hypoxic episode
Partial prolonged ischemia	Cerebral intervascular watershed areas	Prolonged, moderate/or intermittent
Mixed injury	Deep nuclei/cortex and watershed areas	Severe, relatively brief. May be prolonged.
Type 1 cystic encephalomalacia	Cerebral cortex, white matter sparing the basal nuclei	Severe prolonged anoxia
Type 2 cystic encephalomalacia	Cerebral cortex, white matter as well as basal nuclei	Severe, with acute profound anoxia
*Cerebral* = Cortex and subcortical/central white matter involvement, especially parasagittal/watershed territory. *Deep nuclei* = Thalamus, Putamen, ±Caudate nucleus. *White matter* = Periventricular and central cerebral white matter.		

HIBI, hypoxic ischemic brain injury.

**FIGURE 2:**
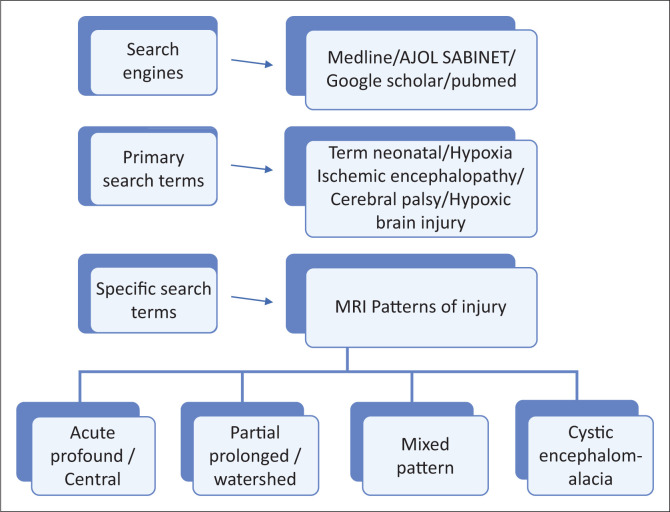
Contextual framework of the narrative review process for term neonatal hypoxic ischemic encephalopathy and the resultant neuroimaging patterns.

A literature review was undertaken and included an electronic search of English language articles on cerebral palsy published between January 2000 and December 2019 on the electronic databases of Medline, Google Scholar, PubMed, African Journals Online (AJOL) and SABINET (South African Bibliographic Information Network). There is a significant difference in the availability of published literature from developed countries versus developing countries including sub-Saharan Africa. In this review, primary searches were performed using keywords such as ‘hypoxia/hypoxic, ischaemic/ischemic, neonatal, encephalopathy, brain injury, magnetic resonance imaging (MRI) and cerebral palsy’. Specific secondary searches included the terms acute profound, central, partial prolonged, total anoxia, asphyxia and cystic encephalomalacia. In addition to peer-reviewed literature, further articles were selected based on manual searches and cross references of cited key articles. Figure 2 shows the framework of the review process followed in the literature search.

A review of Google Scholar for the selected period reveals a total of 545 articles in the English literature using the search terms ‘hypoxic ischemic encephalopathy/magnetic resonance imaging/acute profound/partial prolonged/cystic encephalomalacia’. On SABINET and AJOL, a total of 77 and 13 articles were returned, respectively, on searching for hypoxic ischemic encephalopathy. There is relatively little published in the African literature looking at the MRI features of HIBI or correlation of neuroimaging with perinatal HIBI. One such article is a pictorial review by Dekker et al.,^15^ which described the patterns of brain injury identified on neuroimaging in term and preterm neonates within utero or perinatal hypoxia.

Variable incidences have been reported of these patterns of injury. For instance, a two-centre North American study^16^ performed at the University of California San Francisco (UCSF) and Loma Linda University Children’s Hospital (LLUCH) showed in 173 neonates with encephalopathy, an incidence of watershed pattern/partial prolonged ischemic injury of 45% and basal ganglia/acute profound ischemic pattern in 25%. A comparative European study^17^ performed in the Netherlands analysed MRI studies in 2587 patients with neonatal encephalopathy and showed 104 patients had neuroimaging patterns of HIBI. The acute profound subtype was seen in more than 20% and cystic encephalopathy subtype in approximately 10% of these 104 children with cerebral palsy.

De Vries and Groenendaal^13^ also described the two main forms of HIBI as the basal-ganglia-thalamus and watershed patterns with the former seen in cases of more severe hypoxia. Each of the MRI manifested subtypes of HIBI is associated with clinically variable outcomes and degrees of severity. Retrospective correlation with recorded clinical findings and the overall perinatal scenario including blood tests, Apgar scores and neonatal measurements are key in demonstrating probable etiopathogenesis. Important clinical criteria for diagnosis of neonatal hypoxic encephalopathy as well as Sarnat and Sarnat scoring systems are utilised in the grading of HIBI and neonatal encephalopathy. By a calculated score, the severity of HIBI is estimated and the neonate is classified as having sustained mild, moderate or severe hypoxic brain injury. Congruent to the severity of the hypoxemia and duration thereof will be the emergent pattern of neuroimaging abnormalities detected by MRI sequences. The protocol varies at each centre but a basic list of sequences is included in Table 2.

**TABLE 2:** List of magnetic resonance imaging sequences in the scanning protocol at the principal author’s institution.

**MRI Sequence Protocol for evaluation of hypoxic brain injury**
Sagittal T1-weighted – volumetric
Axial and/or coronal T2-weighted
Diffusion weighted imaging
Susceptibility weighted imaging
Coronal inversion recovery – Volumetric
Coronal inversion recovery – Temporal lobe angulated
Axial T1-weighted
Axial FLAIR – after 6 months of age
MR Spectroscopy in early neonatal period (where available)

MRI, magnetic resonance imaging; FLAIR, fluid attenuated inversion recovery.

## Acute profound ischemia

**FIGURE 3:**
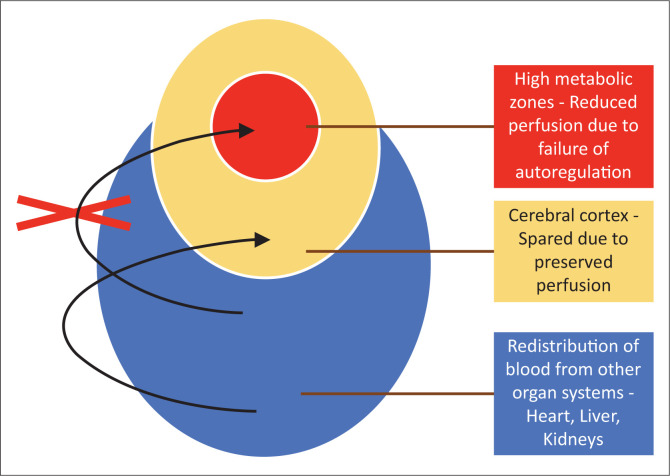
Diagrammatic representation showing redistribution of visceral blood supply to the brain, but failure of cerebral autoregulatory mechanisms to redirect perfusion to the high metabolic zones of the brain.

In the setting of acute cessation of perfusion with rapid progression (e.g. in abruptio placentae), there is insufficient time for the cerebral autoregulatory mechanisms to adequately redirect blood flow to the high metabolic areas of the brain. These are areas of the brain that are actively myelinating with high N-methyl-D–aspartate (NMDA) receptor presence. The failure to protect these areas will result in a primarily central injury pattern with selective neuronal necrosis. This pattern of injury shown in Figure 3 involves the deep basal nuclei (especially the posterior putamina), thalami (particularly the ventrolateral nuclei), perirolandic sensorimotor cortex at the frontal/parietal lobe interface, hippocampal formations and, in some cases, the upper dorsal brainstem and anterior aspect of the superior cerebellar vermis (see list in Table 3). This subtype is also referred to in the literature by the terms central type injury or basal ganglia-thalamus pattern.

**TABLE 3:** High metabolic zones of the brain with highest concentration of N-methyl-D-aspartate receptors.

Perirolandic sensorimotor strip
Thalami (ventral posterior lateral nuclei)
Lentiform nuclei (posterior putamen)
Hippocampi and parahippoocampal gyri
Optic radiation
Heschl’s gyrus at primary auditory cortex
Anterior superior cerebellar vermis
Tegmentum of midbrain and pons

**FIGURE 4:**
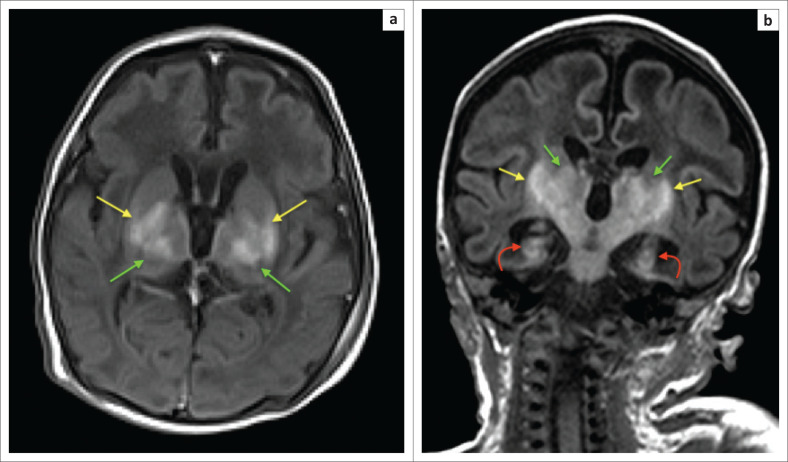
Two-week-old neonate born with grade 3 hypoxic ischemic brain injury and neonatal seizures. (a) An axial T1-weighted sequence showing signal shortening at the dorsal putamina (yellow arrows) and ventral thalami (green arrows). (b) Note, in addition to the basal ganglia and thalamic changes, the hyperintense signal and volume loss at both hippocampi (red arrows).

**FIGURE 5:**
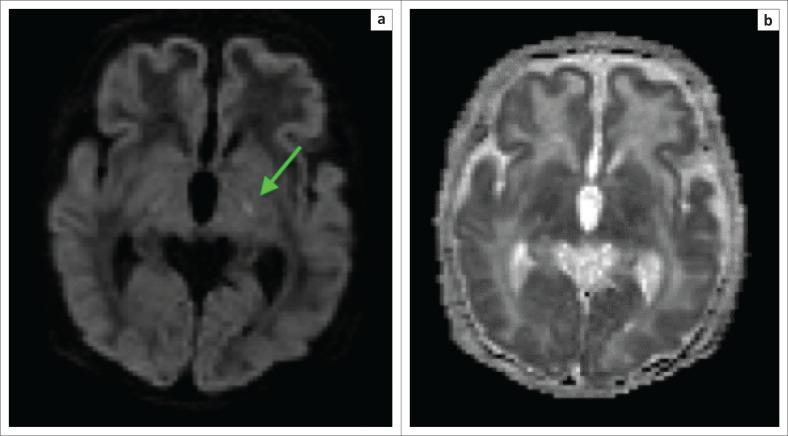
The same child as in Figure 4. (a) The B1000 sequence and (b) the apparent diffusion coefficient (ADC) map obtained after 1 week demonstrating pseudonormalisation phenomenon with trace remaining hyperintensity at the ventral thalamus (green arrow) and no associated ADC shortening.

**FIGURE 6:**
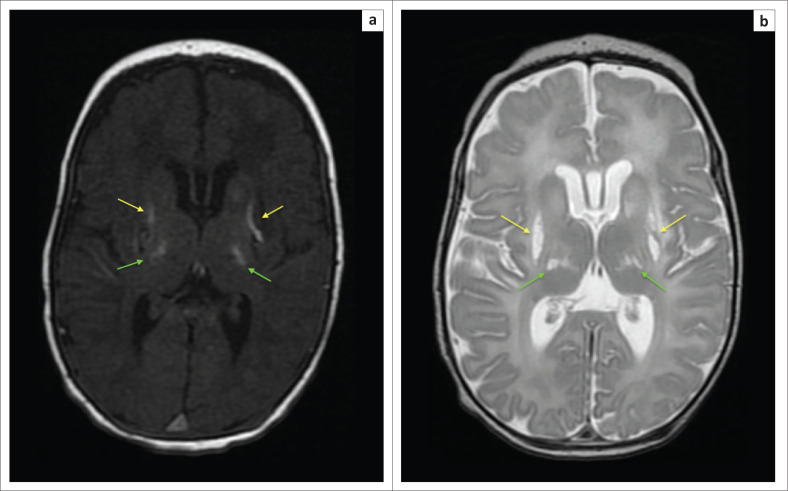
Twenty-seven-day-old male child delivered after abruptio placentae. Axial magnetic resonance imaging images at the level of the basal ganglia demonstrating bilateral fairly symmetrical dorsal putaminal (yellow arrows) and ventral thalamic (green arrows) hyperintensity because of T1 shortening (a) and corresponding T2-weighted hyperintensity and established atrophy of these structures (b). Note that the changes have evolved within the first month of life.

In cases of HIBI, MRI performed in the first 3 days of life will demonstrate little, if any, change on the T1- and T2-weighted conventional sequences.^18^ Diffusion-weighted imaging in the neonate will, however, reveal restricted diffusion in the affected high metabolic zones with no associated paramagnetic signal loss on susceptibility weighted imaging (SWI) sequences. In the latter half of the first week of life, T1-weighted hyperintensity may be seen at the ventral thalamus and dorsal putamina (see Figures 4–6) along with loss of the expected T1-weighted hyperintense line of the posterior limb of the internal capsule in term neonates, known as the absent posterior limb sign. After the first week, the diffusion signal may become falsely negative and this is known as the pseudonormalisation phenomenon (see Figure 5). In addition, it has been shown that diffusion signal abnormalities may re-appear in areas adjacent to foci of pseudonormalisation as the injury evolves in subsequent weeks or early neonatal life.^18,19^

Magnetic resonance spectroscopy has also been shown to be of value in demonstrating HIBI prior to signal changes on the other conventional sequences^19^ as well as pre-empting diffusion changes in a few cases.^20^ A decrease in N-acetylaspartate (NAA) and choline peaks and prominent lactate doublet peaks correlates with areas of selective neuronal necrosis. In addition, reduced NAA/choline or NAA/Cr ratios associated with increased Lac/NAA ratio is predictive of a poor neurodevelopmental outcome.^13^

**FIGURE 7:**
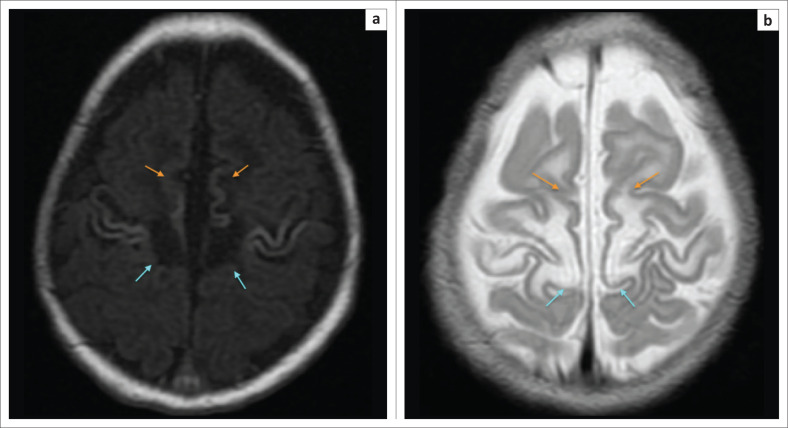
The same child as in Figure 6. Axial magnetic resonance imaging at the superior cerebral convexity demonstrating perirolandic cortical ribbon hyperintensity with T1 shortening (a) on both sides of the central sulcus and T2-weighted hyperintensity of the surrounding sensorimotor cortex (b). Note associated localised parasagittal cortex (orange arrows) and paracentral lobule (cyanide arrows) involvement.

**FIGURE 8:**
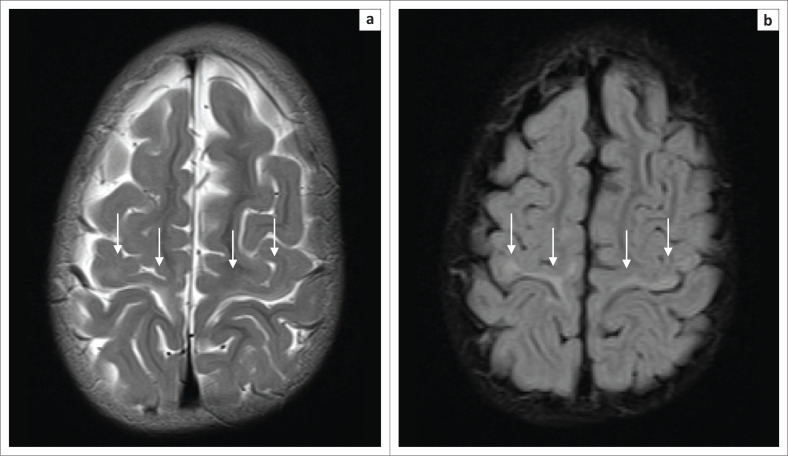
Three-year-old male child with history of neonatal encephalopathy, low Apgar scores and seizures. (a) An axial T2-weighted sequence image, which does not demonstrate the perirolandic changes adequately, and these changes would be difficult to diagnose without the axial FLAIR sequence image (b) which shows that the changes are almost exclusively involving the precentral gyrus.

**FIGURE 9:**
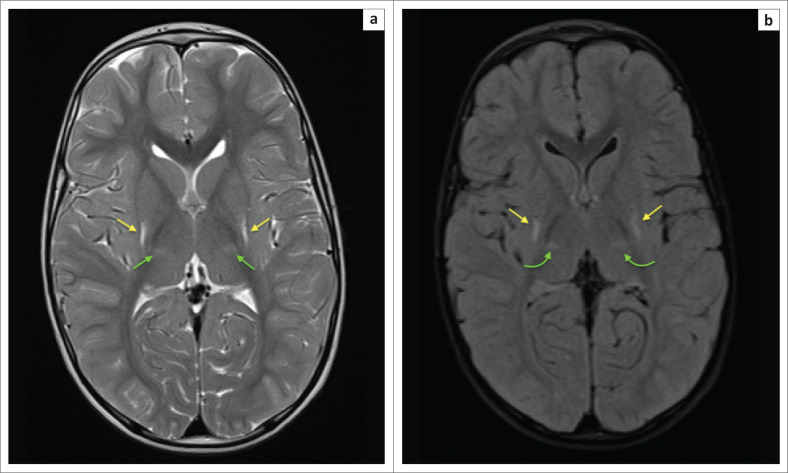
Four-year-old male child with cerebral palsy. Neonatal history of hypoxic ischemic brain injury-related encephalopathy, seizures, requiring ventilation and prolonged 4 week stay in ICU. (a) An axial T2-weighted sequence and (b) an axial FLAIR sequence image showing subtle flame shaped dorsal putaminal (yellow arrows) hyperintensity and smudge-like ventral thalamic (green arrows) hyperintensity bilaterally.

**FIGURE 10:**
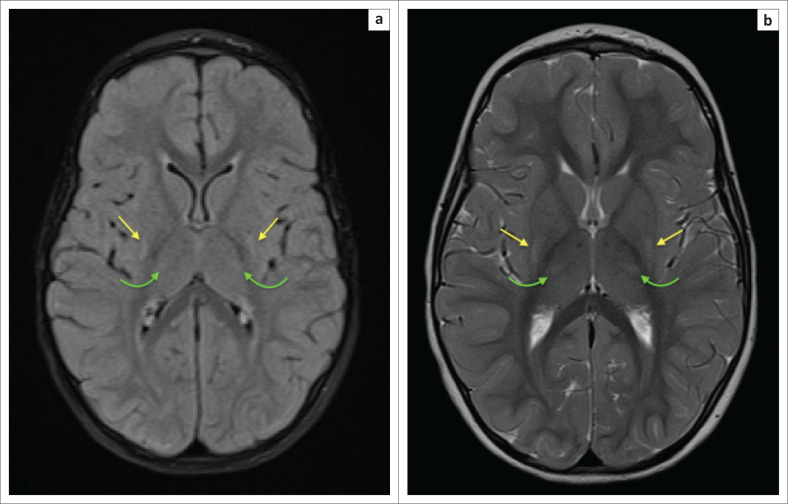
A 2-year-old female child with dystonic cerebral palsy. (a) An axial FLAIR sequence image and (b) an axial T2-weighted image demonstrating even more subtle signal abnormalities at the dorsal putamina (yellow arrows) and ventral thalami (green arrows). These changes can be very difficult to detect and may be omitted by the unsuspecting reporter.

A T1-weighted hyperintense signal outlining the perirolandic cortex is an important feature of acute profound hypoxic injury in the neonate in the latter half of the first month of life (see Figure 7). Subsequent follow-up MRI studies in later childhood reveal persistent T2-weighted and FLAIR hyperintensity in the affected substrate. The degree of signal abnormality seen is extremely variable and can be quite subtle in some instances. In particular, the perirolandic sensorimotor cortex signal change may be limited to one lip of the fronto-parietal cortex interface (see Figure 8). Often, this subtle injury may not be well appreciated on the T2-weighted sequences and is better delineated on axial FLAIR sequences. The dorsal putaminal signal changes may be pin-point sized, and also the ventral thalamic changes may be less obvious (see Figures 9 and 10).

**FIGURE 11:**
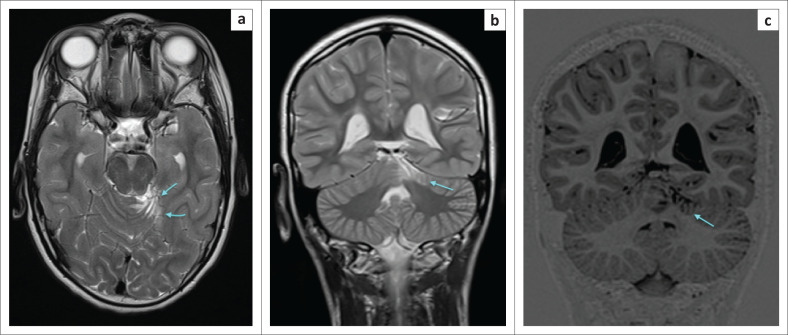
A 9-year-old male child who suffered grade 3 hypoxic ischemic brain injury with Apgar scores of zero at 1 min and 7 at 5 min. (a) An axial T2-weighted, (b) a coronal T2-weighted and (c) a coronal inversion recovery (IR) sequence image demonstrating the left superior cerebellar cortex injury (cyan blue arrows) with localised atrophy involving the left half of the quadrangular lobule. This is an important review area, as in this case, the injury was omitted by the first reporter.

**FIGURE 12:**
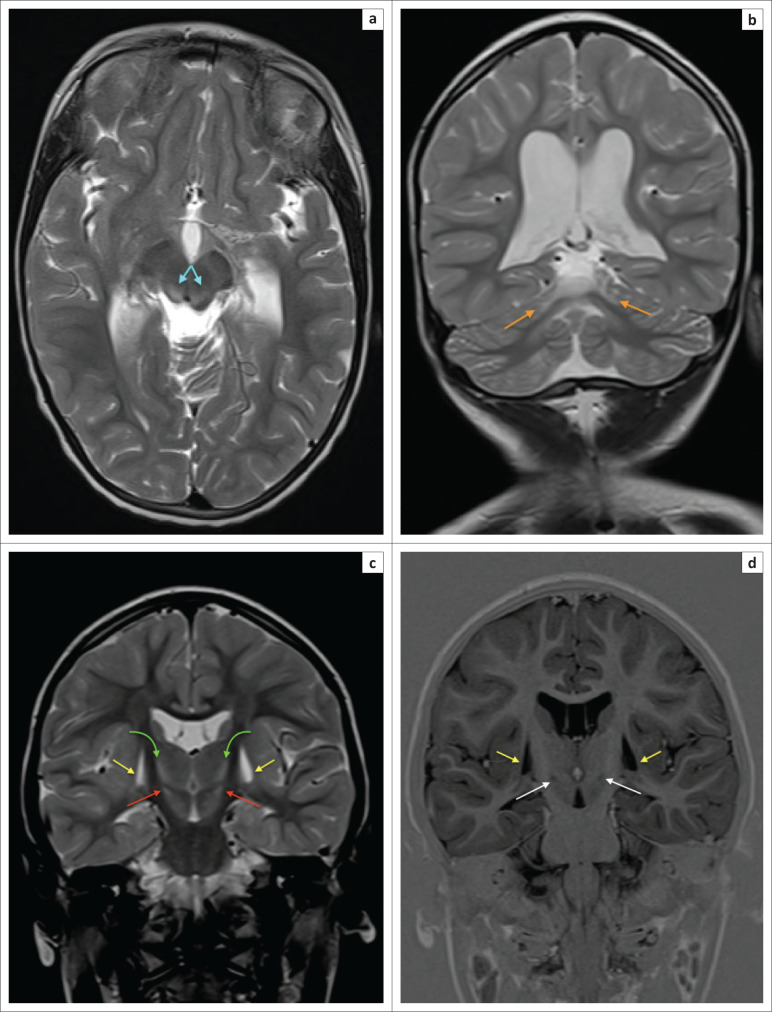
(a) and (b) are T2-weighted images in a 3-year-old female child with acute profound HIBI. (a) Dorsal midbrain tegmentum (cyan blue arrows) signal changes and (b) superior cerebellar (orange arrows) injury. (c) and (d) are coronal T2-weighted and IR sequences in a 7-year-old female child who presents with dyskinetic cerebral palsy after grade 2 hypoxic ischemic encephalopathy. (c) Hyperintensity at the subthalamic nucleus (red arrows) of the upper midbrain and (d) corresponding low signal intensity (white arrows) extending close to the periaqueductal gray (yellow arrows indicate putamen and green arrows indicate thalamus).

Post-hypoxic changes may also be identified in relation to the brainstem and cerebellum. In particular, injury may be seen at the mesencephalo-pontine tegmentum as well as the central tegmental tracts of the pons. Symmetric central tegmental tract hyperintensity is a non-specific finding seen in children with cerebral palsy.^21^ It has been reported as a normal variation in some cases^22^ or attributed in the literature to metabolic and congenital causes. These brainstem and cerebellar changes are exceedingly rare and may be difficult to appreciate on conventional axial sequences. Coronal plane imaging is valuable as shown in Figures 11 and 12 to demonstrate the brainstem and cerebellar changes. Note the propensity for subthalamic nucleus injury,^23^ especially in children with dyskinetic cerebral palsy (as shown in Figure 12c and d). This group of subtle radiological biomarkers may be generally under-reported.

### Pearls of imaging…

*Look for focal or flame-shaped dorsal putaminal and ventral thalamic hyperintensity*.

*The central sulcus may be a site for hidden subtle perirolandic changes*.

*Dorsal brainstem and superior cerebellum are important review areas.The subthalamic nuclei may also show hyperintensity in dyskinetic cerebral palsy*.

*Don’t forget to check the hippocampi*.

## Partial prolonged ischemia

**FIGURE 13:**
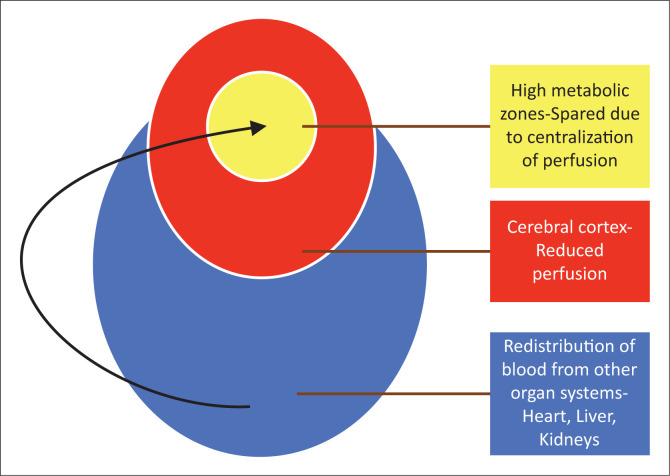
Diagrammatic representation of the redistribution of blood away from the visceral organ systems and cerebral cortex in favour of the most important central brain structures.

**FIGURE 14:**
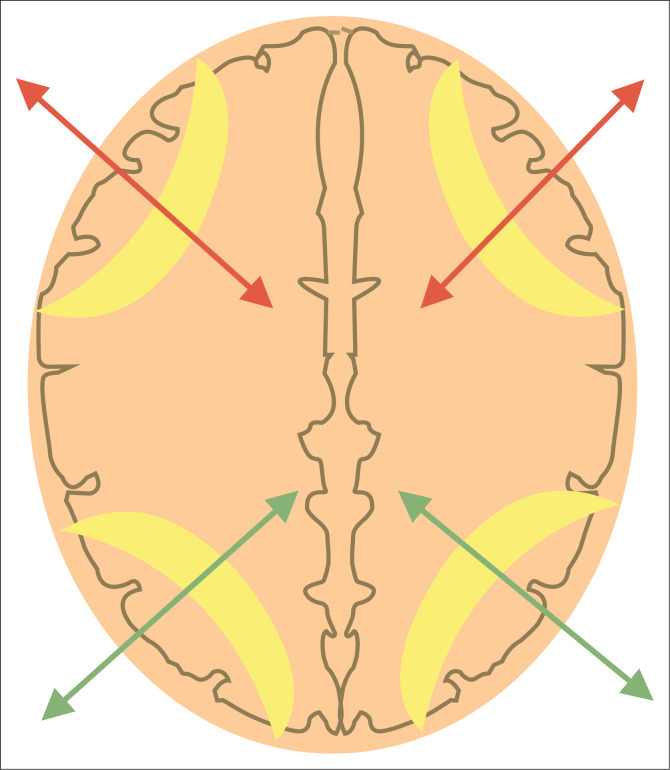
Schematic representation of the interarterial external watershed regions (depicted in yellow overlay) at the centrum ovale. The red arrows indicate the border zone between anterior cerebral artery and middle cerebral artery territories. The green arrows indicate the border zone between middle cerebral artery and posterior cerebral artery territories.

**FIGURE 15:**
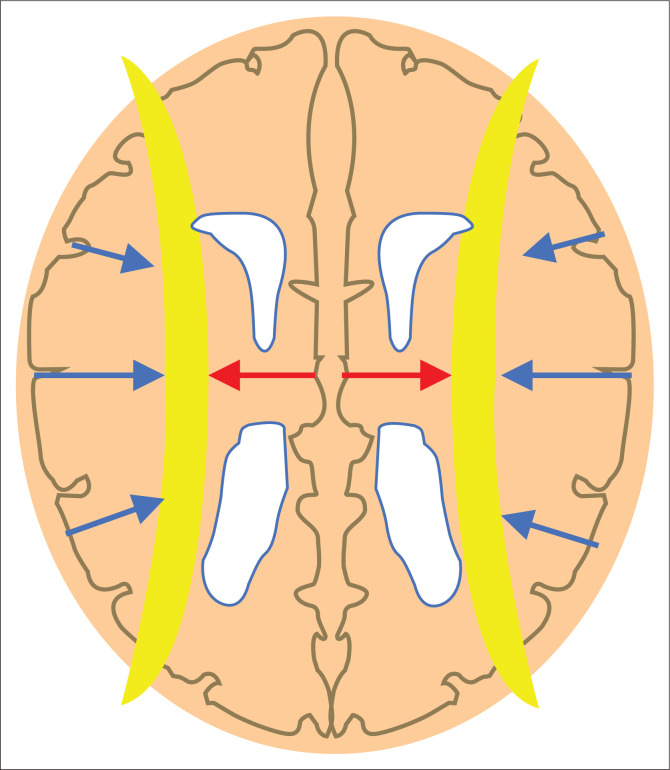
Schematic representation of the internal watershed regions (depicted in yellow overlay). The red arrows indicate the susceptible junction zones of the ventriculofugal vessels coursing away from the ventricles and ventriculopetal vessels coursing inward from the cortex (blue arrows).

When there is mild or moderate hypoxia (e.g. in occult cord prolapse or placental insufficiency), there is sufficient time available for the cerebral auto-regulatory mechanisms to redirect blood flow to the high metabolic areas of the brain, many of which have a greater proportion of NMDA receptors.^14^ This results in sparing of these high metabolic areas at the expense of the watershed areas of the cerebral hemispheres between the major arterial territories, especially at the borders between perfused zones, shown in Figure 13. For term neonates, the external or cortical watershed area is more peripherally positioned at the grey–white matter interface between the major branches of the anterior and posterior circulation^24^ (see Figure 14). The internal watershed or boundary zones are at the parasagittal subcortical white matter secondary to the cerebral arterial tree arrangement with the junction of the ventriculofugal vessels coursing outward from the ventricles and ventriculopetal vessels coursing inward from the cortex (see Figure 15). These boundary zones are susceptible to hypoperfusion injury secondary to autoregulatory shunting of blood to support the high metabolic zones.

**FIGURE 16:**
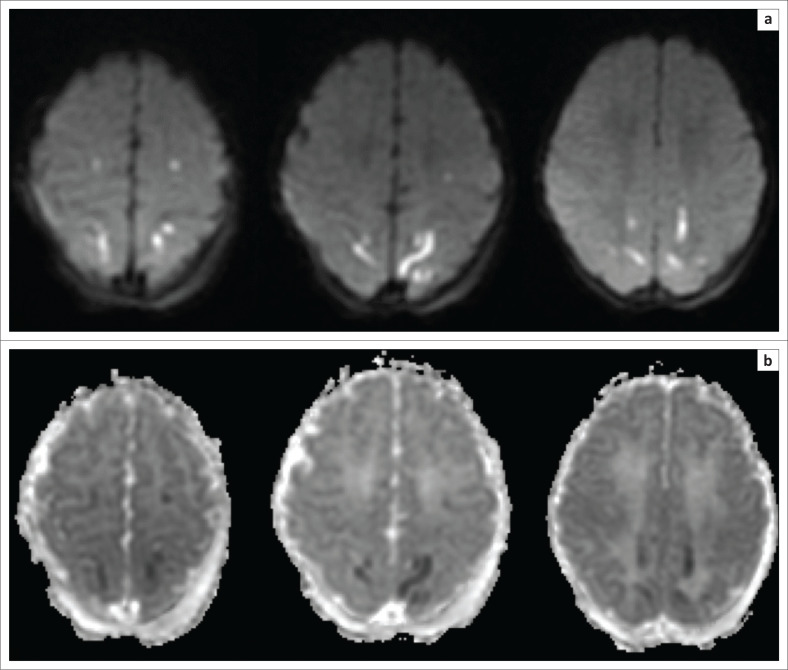
A 2-day-old baby boy presenting with grade 2 hypoxic ischemic brain injury. (a) The diffusion B-1000 trace sequences and (b) the corresponding ADC maps at each level. There are focal areas of restricted diffusion involving the parasagittal watershed zones of both cerebral hemispheres.

**FIGURE 17:**
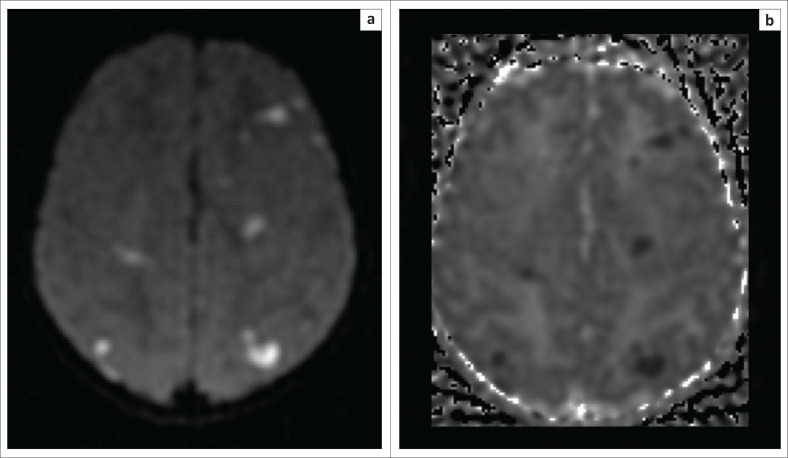
A 3-day-old baby boy with neonatal seizures after grade 2 neonatal hypoxic ischemic brain injury. (a) The diffusion B-1000 trace sequence and (b) the corresponding ADC map. Note that the watershed territory involvement in this child is asymmetric, and this is often seen in partial prolonged ischemia subtype.

**FIGURE 18:**
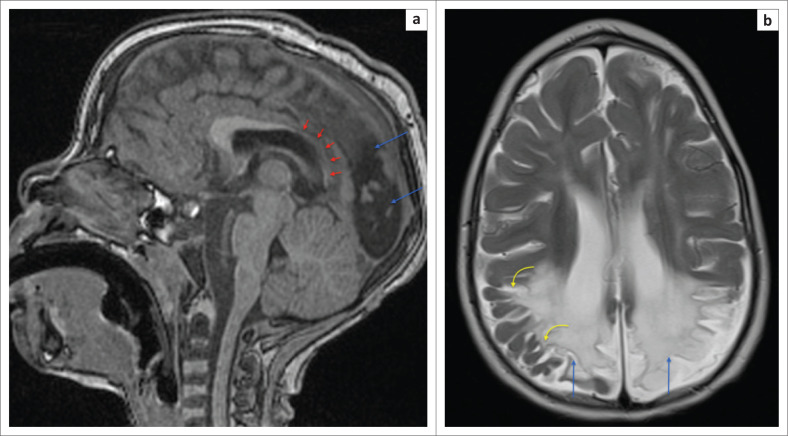
(a) Sagittal T1-weighted image at the midline of the calvarium demonstrating advanced spongiosis of the parietal and occipital lobes (blue arrows) associated with deafferentation thinning of the splenium and isthmus of the corpus callosum (red arrows). Note relatively normal calibre anterior body and genu of corpus callosum. (b) Axial T2-weighted image showing severe biparietal atrophy with ex vacuo dilatation of the lateral ventricles and evidence of ulegyria (yellow arrows). Note less marked changes in the frontal lobes.

**FIGURE 19:**
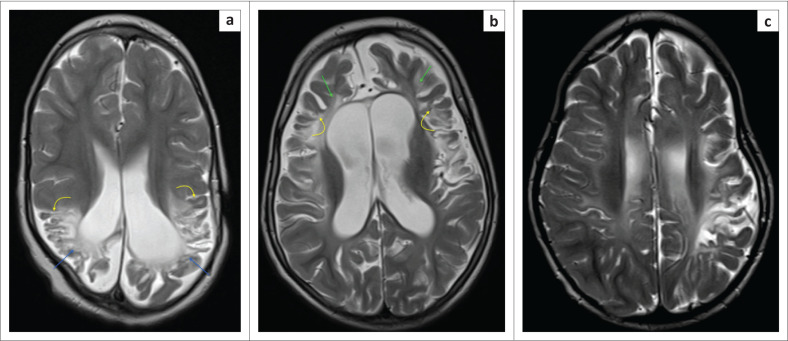
Axial T2-weighted magnetic resonance images demonstrating severe post-hypoxic injury because of partial prolonged ischemia in three different patients. Note the spongiosis of the parietal lobes (blue arrows) (a) and frontal lobes (green arrows) (b) associated with ulegyria (yellow arrows) and secondary ventriculomegaly. (c) The level of centrum ovale demonstrating asymmetric hypoxic ischemic brain injury changes, greater in the left frontal and parietal lobes. This latter pattern of asymmetric involvement is not an uncommon finding.

**FIGURE 20:**
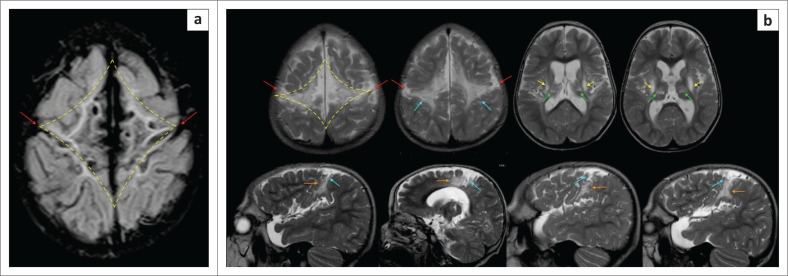
Two examples of parasagittal cerebral injury in children who suffered sentinel events of placental abruption leading to acute profound ischemia (API). (a) This figure shows a diamond-shaped expansion of the frontoparietal parasagittal surface convexity, widening the interhemispheric fissure and tapering bilaterally at the far edge of each central sulcus (red arrows). (b) This figure reveals similar broadening of the extra-axial space because of parasagittal cortex and paracentral lobule injury. Note the contiguous corticospinal tract Wallerian degeneration (orange arrows), the subsiding centrum ovale scaffold (cyan blue arrows) as well as API-related basal ganglia (yellow arrows) and thalamic (green arrows) injury.

As with the APA pattern, in the neonate with PPI, in the first few days of life, there will be restricted diffusion in the affected parasagittal cortex and subcortical white matter at the border zones, shown in Figures 16 and 17. Note that the early diffusion changes may be asymmetric as will be the consequent cerebral encephalomalacia and atrophy (see Figure 20). After a few days, T1-weighted shortening and subsequently T2-weighted hyperintensity is seen at the watershed areas. With progressive evolution of the injury, there is variable cortical thinning demonstrated particularly at the bottom of the sulci, which are more susceptible to ischemia because of the pial vascular supply having an external to internal direction of flow. This leads to pinching off at the base of the sulci with mushroom-shaped gyri known as the ulegyria phenomenon, a specific sign for ischemia.^25^ Secondary features also evolve contiguous to the affected areas of cerebrum with deafferentation thinning of segments of the corpus callosum, hyperintensity and thinning of long tracts because of Wallerian degeneration and ex vacuo dilatation of the lateral ventricles, especially of the trigones and occipital horns (see Figures 18 and 19).

### Pearls of imaging…

*Bilateral medial frontal, parietal and occipital watershed injuries may be asymmetric*.

*Look out for mushroom-shaped gyri of the ulegyria phenomenon*.

*Note secondary features of corpus callosum thinning and ex vacuo dilatation of ventricles*.

Some authors have debated the pathogenesis of the parasagittal cerebral injury, which is often seen in children with HIBI. According to Myers study^26^ and in Volpe et al.,^27^ parasagittal cerebral injury is characteristic of the full-term infant with perinatal asphyxia. The precise pathological evolution of parasagittal injury in the new-born is not known but atrophic gyri, ulegyria or both are the chronic neuropathological correlates. These parasagittal injuries are described at the overlapping and contiguous watershed zones between named vascular territories including the anterior watershed (between the anterior and middle cerebral artery territories), the perisylvian watershed and the posterior watershed (between the posterior and middle cerebral artery territories).^24^ We have however found that injury to the parasagittal cortex (of predominantly the frontal and parietal lobes) may also be because of severe acute profound HIBI. In API parasagittal injuries, there is involvement of the perirolandic cortex as well as the paracentral lobule and the subsequent Wallerian degeneration, which leads to subsidence of the parasagittal mantle. This may result in an inverted V-shaped or diamond-shaped pattern of superior fronto-parietal injury.

In effect, parasagittal injuries probably represent a continuum of hypoxic brain destruction, which may be consequent on API or PPI dependent upon the clinical scenario and presence of primary or exacerbating sentinel events among other feto-maternal factors. Injury may be limited to the perirolandic area (in the case of typical API as in Figures 8 and 9) or may be more extensive in more severe forms of API, extending beyond the central sulcus (as in Figure 20). If the hypoxic episode is more prolonged, cerebral destruction more remote from the central sulcus may ensue resulting in a PPI pattern of injury. Certainly, this spectrum of injury may be seen in a combination of these pathophysiological processes (API and PPI as in Figure 22), and this leads us to a discussion on the mixed subtype of HIBI.

## Mixed pattern

**FIGURE 21:**
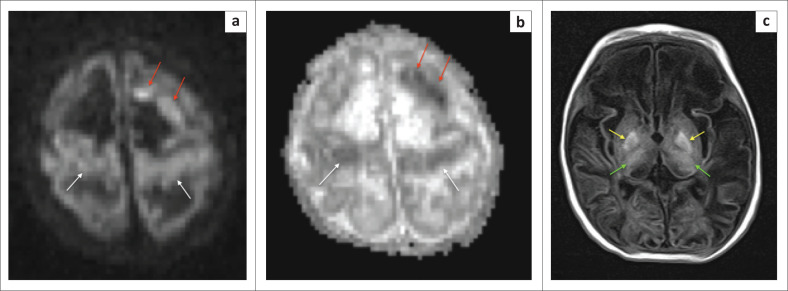
A 15-day-old female child born after abruptio placentae with poor Apgar scores of 0/10 and 1/10 at 1 and 5 min, respectively. Head and body cooling were instituted but the clinical condition remained poor with seizures. The diffusion sequence (a) shows resolving diffusion signal abnormality (white arrows) at the perirolandic cortex and paracentral lobules bilaterally but there is persistent restricted diffusion at the left frontal lobe (red arrows) with ADC shortening in (b) because of watershed zone ischemia. (c) Note the established ischemic injury of the thalami (green arrows) and lentiform nuclei (yellow arrows) with T1-weighted hyperintensity. This is in keeping with evolving mixed subtype of hypoxic ischemic brain injury in the early neonate.

**FIGURE 22:**
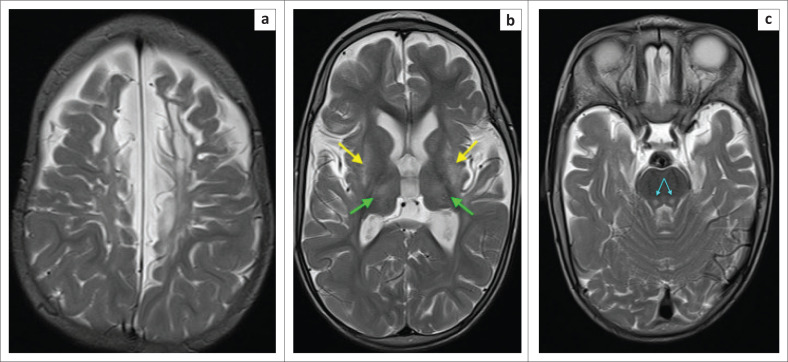
A 2-year-old male child with severe mixed type hypoxic ischemic brain injury. T2-weighted axial images (a) at the level of the centrum ovale demonstrating parasagittal frontal lobe injury with atrophy beyond the central sulcus and paracentral lobule, involving the superior frontal gyri with ulegyria, (b) at the level of the basal ganglia shows the hyperintense signal change at the dorsal putamina (yellow arrows) and ventral thalami (green arrows) and (c) at the level of the superior cerebellar peduncles reveals prominent central tegmental tract (cyan blue arrows) hyperintensity at the dorsum of the pons.

**FIGURE 23:**
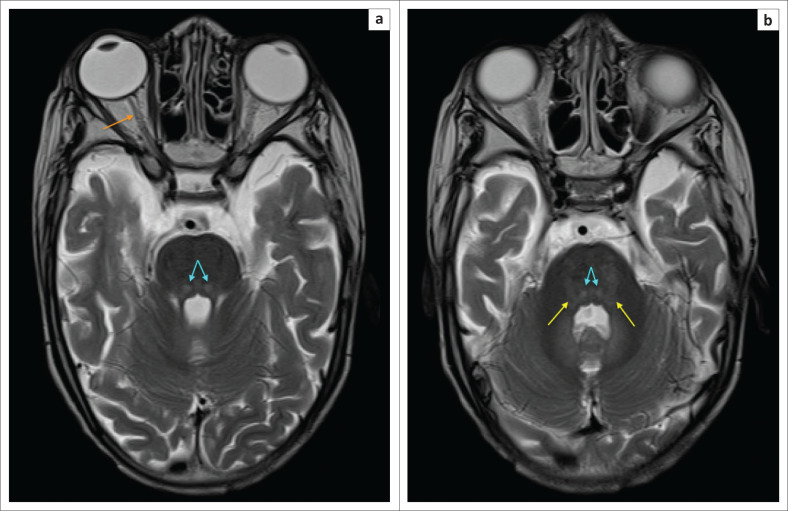
A 7-year-old male child with mixed subtype hypoxic ischemic brain injury. Axial T2-weighted sequence images through the midbrain and pons show dorsal tegmental (yellow arrows) and central tegmental tract (cyan blue arrows) hyperintensity. Note severe cerebral atrophy and optic atrophy (orange arrows).

**FIGURE 24:**
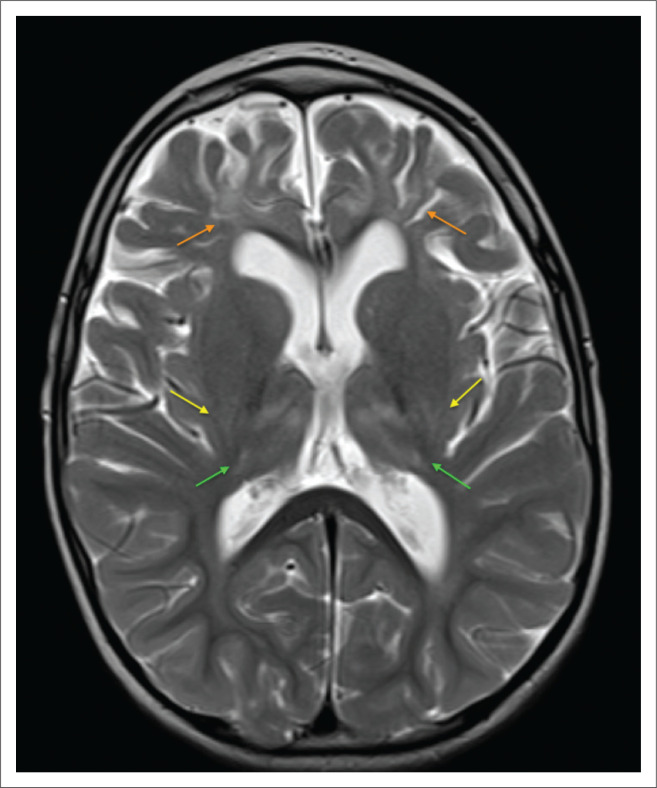
Axial T2-weighted image at the level of the basal nuclei performed on a 7-year-old male child. There is bifrontal encephalomalacia involving the middle and inferior frontal gyri (orange arrows) with ulegyria. Subtle hyperintensity is present at the posterior tips of the putamina (yellow arrows). Note in addition, loss of volume and more extensive hyperintensity of both thalami (green arrows). The combination is indicative of a mixed pattern of cerebral injury.

In some cases, a period of prolonged poor perfusion or hypoxemia (e.g. recurrent antepartum haemorrhage because of placenta praevia) may be followed by a further sentinel event (e.g. in setting of type B nuchal cord) leading to acute profound hypoxic brain injury superimposed on a background of watershed zone partial prolonged asphyxia. This pattern of injury is not uncommon in the South African population and is being more frequently reported in our cases of perinatal hypoxic ischemic encephalopathy. There is a combination of MRI findings referable to the basal nuclei, thalami, perirolandic cortex in addition to the variable watershed or parasagittal border zone injuries. In the acute postnatal period, diffusion signal abnormalities will be seen affecting both the watershed areas and high metabolic structures. Some days later, there may be discordant diffusion signal changes with some areas showing normal diffusion signal, whereas there may be diffusion restriction with ADC shortening in other areas as shown in Figure 21. Based on the degree of hypoxic injury to the watershed zones, there may be isolated ulegyria, localised or asymmetric cerebral atrophy, areas of necrosis or cystic encephalomalacia (Figures 22–24). Brainstem injury (Figures 22 and 23) and cerebellar changes may also be seen, and these are usually correlative with the acute profound ischemic component of this mixed pattern of injury.

## Multilobar cystic encephalomalacia/multicystic encephalopathy

**FIGURE 25:**
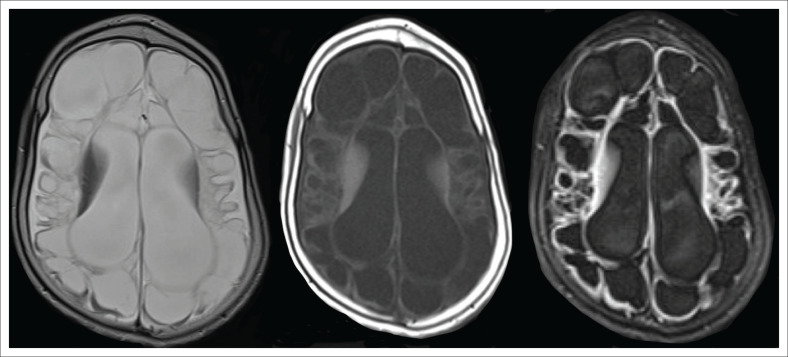
A 3-year-old male child with spastic cerebral palsy and blindness. Recorded neonatal encephalopathy with low Apgar scores and neonatal seizures. Axial T2-weighted, T1-weighted and FLAIR sequence images show extensive cerebral destruction with grey and white matter injury, cystic encephalomalacia and ex vacuo dilatation of the lateral ventricles.

**FIGURE 26:**
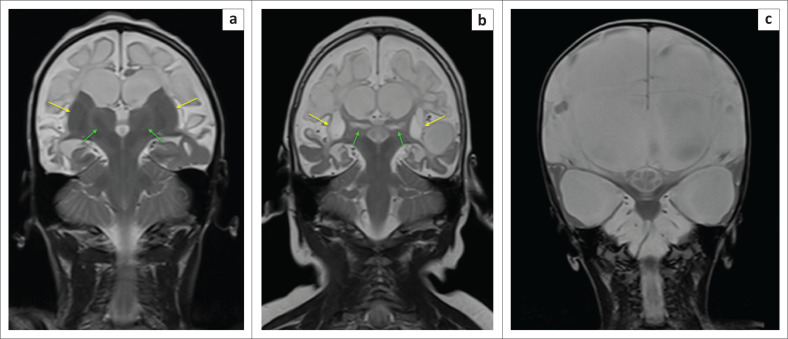
The spectrum of basal ganglia involvement in association with cystic encephalomalacia shown on coronal T2-weighted images in three children. (a) A 4--year-old female child with complete sparing of the basal ganglia, thalami and cerebellum. (b) A 2-year-old male child with necrosis of the putamen (yellow arrows) and ventral thalamus (green arrows) bilaterally. (c) A 4-year-old-male child who suffered severe total anoxia related to abruptio placentae, with birth weight of 3.35 kg and Apgar scores of 1/10 and 3/10. Note near-complete cerebral cystic encephalomalacia, atrophy of lentiform nuclei and severe thalamic destruction.

In very severe cases of hypoxia or total anoxia, HIBI can extend beyond the watershed or boundary zones to involve major vascular territories completely. The cerebrum becomes infarcted more globally and diffusion signal abnormality may be difficult to appreciate as the entire cerebrum can show restricted diffusion with relatively no normal cerebral lobe to compare with. Comparison of the abnormal cerebral diffusion signal with the normal cerebellum is a useful tool to confirm the ‘White Cerebrum’ sign. As this injury evolves, there is progressive loss of cerebral volume with extensive irreversible neuronal destruction, vacuolation, cystic change or spongiosis. Multilobar cystic encephalomalacia (as in Figure 25) is the result. There is usually marked ventriculomegaly because of ex vacuo dilatation from the extensive surrounding white matter destruction. Relative sparing of the immediate periventricular white matter and the basal nuclei may be seen. This is classified as the first subtype of cystic encephalomalacia, which is probably an exaggerated form of PPI (as in Figure 26a). In cases where the insult was an acute, severe and sustained hypoxic event, the basal nuclei are more likely to be involved, presenting with the second subtype of cystic encephalomalacia. Depending on the severity of the hypoxia and the autoregulatory response, a variable degree of basal ganglia involvement will be seen (as seen in Figure 26b and c).

With regard to the multicystic encephalopathy or cystic encephalomalacia group, noting that there are several causes of this pattern of injury, we propose two subtypes that would result from hypoxic ischemic injury depending on the timing and severity of the insult. In most such cases, the entire cerebrum is affected, only sparing some portions of the temporal lobes. The key distinguishing feature is the involvement of the basal ganglia, which indicates probable primary (or superadded) APA injury.

The pattern of multicystic encephalopathy when demonstrated on an MRI study performed several years later is a non-specific pattern of cerebral injury and can represent the result of late in utero or perinatal ischemia, infection or metabolic abnormalities. With the increased use of foetal MRI, in utero causes of multicystic encephalomalacia may be more easily detected and these can be distinguished from perinatal HIBI. In cases where this pattern is present in utero, most of the insults would have occurred prior to the 28th week of gestation leading to hydranencephaly or porencephaly. The pathophysiology of the cystic pattern in a term infant, however, has an underlying common factor of severe cerebral hypoxia, linking all possible aetiologies. These include foetal transfusion syndrome in monozygotic twin pregnancy, cerebritis (e.g. Citrobacter), meningo-encephalitis (e.g. Herpes simplex 2), severe maternal hypotension (e.g. abruptio placentae) or rarely in postnatal non-accidental injury. One must be mindful of the other possibilities; however, HIBI has been shown to be the commonest cause of this cystic pattern.^28^

## Conclusion

For almost three decades, MRI has been utilised in the evaluation of HIBI in children with cerebral palsy. MRI is a clinically independent valuable biomarker of HIBI in the setting of neonatal encephalopathy. Additionally, as shown in this pictorial essay, MRI can be used to retrospectively correlate the pattern of cerebral injury with possible clinicopathogenesis. Radiologists therefore play a key role in confirming the presence of cerebral injury. Using the proposed simplified classification of the subtypes of injury allows description of the pattern of injury shown on MRI and enables correlation with the perinatal events leading to neonatal encephalopathy. The reporting radiologist who is familiar with these subtypes is therefore well-positioned to lead expert evidence and provide confirmation of the injury pattern in medicolegal cases.
